# Differential Susceptibility of Human Peripheral Blood T Cells to Suppression by Environmental Levels of Sodium Arsenite and Monomethylarsonous Acid

**DOI:** 10.1371/journal.pone.0109192

**Published:** 2014-10-01

**Authors:** Scott W. Burchiel, Fredine T. Lauer, Ellen J. Beswick, A. Jay Gandolfi, Faruque Parvez, Ke Jian Liu, Laurie G. Hudson

**Affiliations:** 1 Department of Pharmaceutical Sciences, University of New Mexico, Albuquerque, New Mexico, United States of America; 2 Department of Molecular Genetics and Microbiology, University of New Mexico, Albuquerque, New Mexico, United States of America; 3 Department of Pharmacology and Toxicology, University of Arizona, Tucson, Arizona, United States of America; 4 Department of Environmental Health Sciences, Columbia University Mailman School of Public Health, New York, New York, United States of America; University of Kentucky, United States of America

## Abstract

Human exposure to arsenic in drinking water is known to contribute to many different health outcomes such as cancer, diabetes, and cardiopulmonary disease. Several epidemiological studies suggest that T cell function is also altered by drinking water arsenic exposure. However, it is unclear how individual responses differ to various levels of exposure to arsenic. Our laboratory has recently identified differential responses of human peripheral blood mononuclear cell (HPMBC) T cells as measured by polyclonal T cell activation by mitogens during sodium arsenite exposure. T cells from certain healthy individuals exposed to various concentrations (1–100 nM) of arsenite *in vitro* showed a dose-dependent suppression at these extremely low concentrations (∼0.1–10 ppb) of arsenite, whereas other individuals were not suppressed at low concentrations. In a series of more than 30 normal donors, two individuals were found to be sensitive to low concentration (10 nM equivalent ∼1 ppb drinking water exposure) to sodium arsenite-induced inhibition of T cell proliferation produced by phytohemagglutinin (PHA) and anti-CD3/anti-CD28. In an arsenite-susceptible individual, arsenite suppressed the activation of Th1 (Tbet) cells, and decreased the percentage of cells in the double positive Th17 (RORγt) and Treg (FoxP3) population. While the majority of normal blood donors tested were not susceptible to inhibition of proliferation at the 1–100 nM concentrations of As^+3^, it was found that all donors were sensitive to suppression by 100 nM monomethylarsonous acid (MMA^+3^), a key metabolite of arsenite. Thus, our studies demonstrate for the first time that low ppb-equivalent concentrations of As^+3^ are immunosuppressive to HPBMC T cells in some individuals, but that most donor HPBMC are sensitive to suppression by MMA^+3^ at environmentally relevant exposure levels.

## Introduction

Exposure of human populations to arsenic via drinking water and diet has been determined to be a major worldwide environmental public health issue [1]. Despite this evidence, there has been inadequate characterization of the effects of arsenic on the immune system of humans. Our laboratory is interested in examining altered immune function produced by ubiquitous environmental agents. Previous studies have shown that arsenite may alter immune function at environmentally relevant levels of exposure in mouse models examined *in vitro* and *in vivo* [2], [3].

There have been a few studies that have examined the effects of arsenic on human T cell immune function in human populations exposed through drinking water. These studies are important because infections, cancer, and other disease may result from immune suppression. Soto-Pena et al. [4] found that phytohemmagglutinin (PHA)-induced T cell proliferation was suppressed in children in Mexico ingesting >100 ppb arsenite (As^+3^). Biswas et al. [5] found that humans exposed to arsenic via drinking water in Bangladesh had suppressed T cell responses to concanavallin A, as well as decreased T cell cytokine production.

In vitro, Gonsebatt et al. [6] found that 1–100 nM concentrations of As^+3^ prolonged the cell cycle of PBMC. More recently, Morzadec et al. [7], [8] found that HPMC exposed to 1–2 µM As^+3^ were suppressed in their anti-CD3/anti-CD28 stimulated T cell proliferation and had selective effects on Th cell subsets. Because arsenite (As^+3^) is metabolized to toxic species *in vivo* such as MMA^+3^ following the action of arsenite 3-methyltransferase (AS3MT), it is also important to examine the effects of this arsenite metabolite on HPBMC to better understand potential mechanisms of immunotoxicty. These studies are significant because the AS3MT enzyme is polymorphic and differentially expressed in humans affecting the overall toxicity of arsenic and its elimination from the body [9], [10].

In the present studies, it was found that low concentrations 1–100 nM range (representing low ppb levels of exposure in human populations) of As^+3^ suppressed the PHA mitogen response of human T cells in a small percentage of donors, whereas 100 nM MMA^+3^ suppressed the response in the majority of donors. It also appeared that the PHA response was more sensitive to suppression by MMA^+3^ than was the anit-CD3/anti-CD28 response, suggesting that macrophages may be important in the response to arsenic exposures.

## Materials and Methods

### Chemicals and Reagents

Sodium arsenite (NaAsO_2_), Dulbecco’s Buffered Phosphate Solution (PBS, without calcium or magnesium), and base medium RPMI 1640 HEPES modified were purchased from Sigma-Aldrich (St. Louis, MO). Tritiated thymidine ([^3^H]-thymidine) was purchased from Perkin Elmer (Waltham, MA).

### Donor Recruitment and HPBMC Preparation

Individuals between the ages of 18 and 50 years of age who were in good health were recruited for this study through University of New Mexico’s (UNM) Clinical and Translational Sciences Center (CTSC) according to our UNM Health Sciences Human Research Review Committee approved protocol. Written consent was obtained from participants who were non-smokers, not pregnant, who were not currently taking any prescribed immunosuppressants or over-the-counter medications that might interfere with an immune response, were consented for the study. At the time of consent blood was drawn for a complete blood cell differential (CBC-Diff) to determine if white blood cell populations were within the reference range and to affirm that the participant is no anemic. If all results for the CBC-Diff were within 10% of the reference range, 100 ml of blood was drawn in heparinized tubes by trained personnel at the CTSC. Human peripheral blood mononuclear cells (HPBMC) were separated according to Davila et al. [11]. Briefly, blood was mixed 1∶1 with Dulbecco’s Phosphate Buffer Solution (DPBS^−^; Sigma-Aldrich, St. Louis, MO) at room temperature. The mixture was then layered over 20 ml Fico/Lite solution (room temperature; Atlanta Biologicals Flowery Branch, GA). The layered blood was centrifuged for 30 min at RT with no break at 400*×g*. The mononuclear cell layer, at the interface of the plasma/DPBS^−^ and the Fico/Lite layers, was collected and the cells were washed twice with cold DPBS^−^ and counted.

### T cell proliferation assay

For mitogenesis, 1×10^5^/well human peripheral blood mononuclear cells (HPBMC) were incubated with 0.1, 1, 10 or 100 nM sodium arsenite (As^+3^) or monomethylarsonous acid (MMA^+3^) in 96-well flat bottom plates coated with anti-CD3 and soluble anti-CD28 (Affymetrix eBioscience, San Diego, CA) or in round bottom 96-well plates for phytohemagglutinin (PHA; Roche, Indianapolis, IN) in a humidified incubator at 37°C with 5% CO_2_. After approximately 54 hrs, cells were pulsed with 1 µCi of [^3^H]-thymidine and incubated overnight. The following day cells were harvested with a Brandell model cell harvester. [^3^H]-Thymidine incorporation was assessed by liquid scintillation counting (LS 6500, Beckman Coulter).

### Intracellular staining of T cell transcription factors

Blood was drawn from a healthy individual as described above. CD4 positive cells were then isolated using EasySep Human Naïve CD4^+^ T Cell Enrichment Kit (StemCell, Vancouver, BC, Canada), which is a negative selection process. Briefly, cells were resuspended to 5×10^7^ cell/ml in DPBS^−^ +2% FBS+1 mM EDTA in 5 ml polystyrene tubes, and the EasySep biotinylated anti-CD45RO antibody was added at 50 µl per ml cells. Cells were mixed and incubated at RT for 15 min. The EasySep human naïve CD4^+^ T cell enrichment cocktail was added at 50 µl/ml cells, mixed and incubated at RT for 10 min. Next the nanoparticles supplied in the kit were added at 100 µl/ml cells, mixed and incubated at RT for 10 min. The tube containing the cells was placed into the magnet and left to rest for 10 min. Cells were poured off into a new container which was placed into the magnet and left to rest for 10 min. Following separation, cells were poured off into a new container, counted and brought up to 1×10^6^ cell/ml. These cells were then activated using Dynabeads Human T-Expander anti-CD3/anti-CD28 beads (Life Technologies, Grand Island, NY) and were divided into treatments and plated for approximately 96 hr. To activate the cells the Dynabeads were first washed following the manufacturer’s directions, after determining the amount of beads needed. An aliquot of cells was removed and incubated with washed M-450 Epoxy Dynabeads and utilized as an unstimulated control. As mentioned above the cells were incubated for approximately 96 hr in a humidified incubator at 37°C and 5% CO_2_. Following activation/treatment cells were harvested into three – 12×75 mm non-sterile, round- bottom, polystyrene tubes and stained using antibodies (Affymetrix, eBioscience, San Diego, CA) against GATA-3, RORγt, Foxp3 and T-bet transcription factors. Isotype controls (Affymetrix, eBioscience, San Diego, CA) with the appropriate fluorophores were also included in this assay. Foxp3 and RORγt staining was done in one tube, GATA-3 and T-bet staining was done in another tube and the isotype control was ran in the third tube. To stain cells, they were first washed with DPBS^−^+1% FBS. One ml Foxp3/Fix/Perm (eBioscience) was added to each tube, tubes were pulse votexed and incubated at RT for 40 min in the dark. Two ml of Permeabilization Buffer was added to each tube. Tubes were centrifuged at 400*×g* for 5 min, the supernatant was aspirated and cells were resuspended in 100 µl of Permeabilization Buffer. Either 2% normal mouse or rat serum was added to tubes, samples were incubated at RT for 15 min to block nonspecific staining. Antibodies were added at the recommended amounts with, isotype controls were incubated with isotype antibodies at matching amounts. Samples were incubated in the dark at RT for 30 min. Two ml of Permeabilization Buffer was added to each tube. Tubes were centrifuged as described above, aspirated, and resuspended in 400 µl Flow Cytometry Staining Buffer. Samples were run and analyzed using the Accuri C6 Flow Cytometer (BD Biosciences, San Jose, CA). Quadrant analysis was used to determine the percentage of cells positive for each transcription factor.

### Urinary Arsenic Speciation Analysis

Urinary arsenic speciation was performed on an Agilent 7700x, using anion exchange column and an ammonium carbonate gradient using methods adapted from Milstein et al. [12]. Briefly, the HPLC system consisted of an Agilent 1260 HPLC with Hamilton PRP-X100 column (10 um, 250×4.1 mm) and guard cartridge. The mobile phase was ammonium carbonate (pH 8.75) with 2% methanol in a gradient, 10 mM for 2 min, ramp to 50 mM over 1 min, and hold for 9 minutes. The column temperature was maintained at 30°C and samples were kept at 4°C in a temperature-controlled autosampler. The ICP-MS with a Micromist nebulizer (Glass Expansion) served as the detector. The operating parameters were as follows: RF power 1550 watts, plasma gas flow 15 L/min, carrier flow 0.85 L/min, 0.15 L/min makeup, and arsenic was measured at 75 m/z in He mode. Quality assurance protocol for As speciation included the use of certified reference materials such as NIST 2669 or INSPQ urine samples or NIST1568b rice flour. For each batch of 30 samples, at least three samples were spiked with a low-to-mid range standard to monitor As recovery for each species.

### Statistical analysis

T-cell proliferation assays using PHA and anti-CD3/anti-CD28 were run in replicates of 6 in each treatment group. The intracellular staining of T-cell subsets were run in replicates of 3 per treatment. Differences between treatment groups were tested by one-way ANOVA or t-test using SigmaPlot software version 12.5 (Systat Software, Inc., Richmond, CA). Error bars indicate the Means ± standard deviation (SD) of the sample set. Bio statistical significance was set at p≤0.05. Box and Whisker plots demonstrate 10th, 25th, 75th, and 90th percentiles. Filled circles indicate outliers; red line is the mean; black line is the median. The urinary arsenic MMA and PHA-induced cell proliferation values were log- transformed to normalize their distributions. We conducted a Spearman correlation between urinary arsenic MMA and PHA-induced cell proliferation using Statistical Analysis Software (SAS 9.3) (SAS Institute Inc.).

## Results

**Figure 1:**
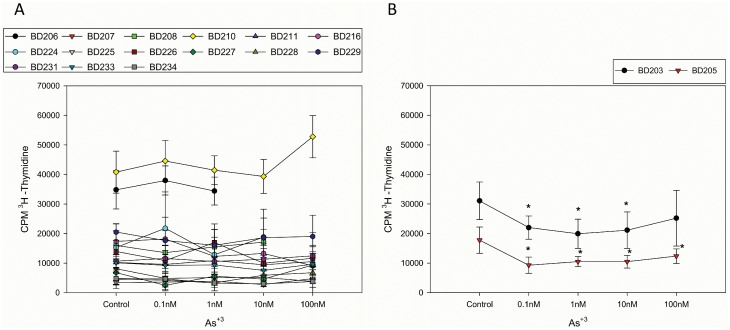
Evaluation of As^+3^ inhibition of HPBMC proliferation. In panel A, the typical response of 15 donors is shown where there is no inhibition of PHA-induced T cell proliferation. In panel B, 2 donors are shown that were extremely sensitive to low dose (0.1–1 nM) inhibition of the PHA response. Data shown are the Mean ± SD with *p<.05.

Because we are interested in the effects of environmentally relevant concentrations (1–300 ppb drinking water and food equivalent) of As^+3^ on human T cell function, a series of experiments were conducted in normal HPBMC to determine the influence of As^+3^ on T cell proliferation. In a series of 17 normal human donors, it was found that *in vitro* exposure to 1–100 nM As^+3^ had little effect on T cell proliferation induced by PHA in 15 donors (Figure 1A). Two individuals were identified who were suppressed by extremely low concentrations (0.1 and 10 nM) of As^+3^ (Figure 1B). This phenotype was observed on multiple occasions in repeat studies. One of these susceptible individuals (BD203) was further examined in a follow-up study.

In accordance with previous studies that have attempted to characterize the target cells associated with T cell suppression [7] by As^+3^, purified naïve CD4 T cells representing approximately 1–2% of total cells from the HPBMC of a sensitive donor (BD203), were stimulated with anti-CD3 and anti-CD28 coated beads. The potential effects of As^+3^ on T cell differentiation into the various known CD4 subsets were determined by flow cytometry analysis of transcription factor expression. Naïve CD4 T cells (98–99% of unstimulated cells seen in % Negative Stain) were identified by lack of expression of the transcription factors for Th1 (Tbet), Th2 (GATA3), Th17 (RORγt), and Treg (FoxP3). As shown in Table 1, anti-CD3/anti-CD28 was found to activate all but 7.5–8.9% of these cells to express some combination of these transcription factors. It is notable that As^+3^ produced a concentration-dependent increase in the number of Negative Staining cells with 14.7% negative staining at 1 nM As^+3^ and 20.6% negative staining in the 10 nM treatment. The results provide an independent measure of the effects of As^+3^ on T cell activation in this sensitive donor.

**Table 1:** Arsenite Suppresses Anti-CD3/Anti-CD28 Naïve HPBMC T cell Differentiation.

	Non-stimulated	Anti-CD3/Anti-CD28	Anti-CD3/Anti-CD28+1 nM As^+3^	Anti-CD3/Anti-CD28+10 nM As^+3^
% Tbet (Th1)	1.44±0.34	82±1.5	79±2.4	74±4.4*
% Gata3 (Th2)	0.0±0.0	0.03±0.02	0.2±0.05*	0.2±0.06*
% Tbet+Gata3 (DP)	0.0±0.0	1.4±0.3	1.0±0.2	0.9±0.07*
% Neg Stain	**98.1±0.3**	**8.9±1.3**	14.7±2.3	20.6±4.3*
% RORγt (Th17)	0.4±0.4	54.9±2.0	58.3±0.6*	57.8±1.8
% FoxP3 (Treg)	0.04±0.03	1.0±0.5	1.6±0.4	2.2±1.3
% RORγt+FoxP3 (DP)	0.01±0.02	36.1±0.6	32.5±0.5*	30.3±2.4*
% Neg Stain	99.5±0.4	7.5±1.0	7.4±0.4	9.3±2.9

Note: DP = double positive; Values shown are the Mean ± SD with *indicating statistical significance at p<.05.

The primary effect of As^+3^ was found to be inhibition of the differentiation of Th1 cells defined by Tbet, which declined from 82% after stimulation with anti-CD3/anti-CD28 to 74% in the 10 nM As^+3^ treatment group. There were very few Th2 GATA3 positive cells detected and therefore it is unclear if As^+3^ effects Th2 cell differentiation without further manipulation of cultures. The Th17 population (defined by RORγt) overlapped with the Treg (FoxP3 expressing) fraction and could only be assessed in this double-positive (DP) population. There was a small decrease from 36.1% (control) in the RORγt/FoxP3 DP population to 30.3% in the 10 nM As^+3^ treatment group. These results appear to be consistent with the previous studies of Morzadec et al. [8] where they found that the Th17 cells were the targets at higher concentrations (1–2 µM) of As^+3^.

**Figure 2:**
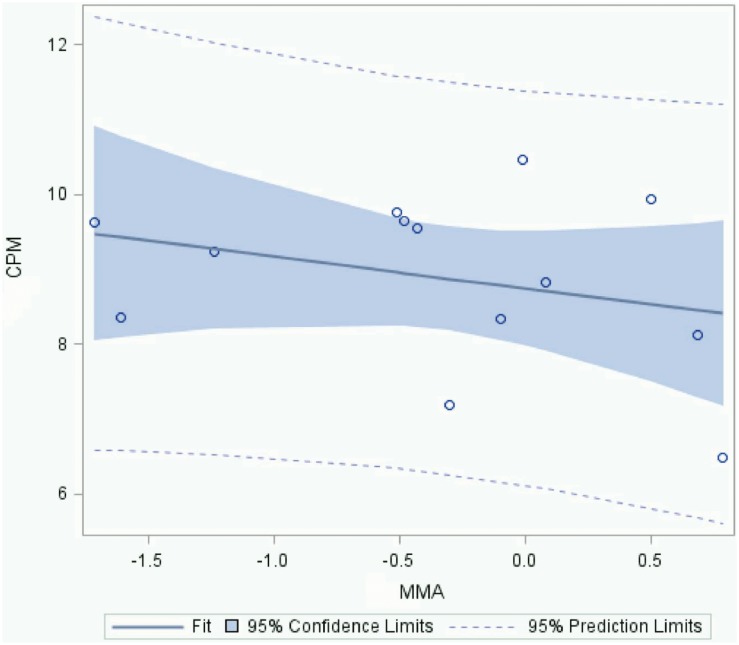
Correlation analysis of MMA in urine with PHA-induced T cell stimulation (^3^H-Thymidine counts per minute (CPM). There is an indication of a weak correlation between urinary MMA (combined MMA^+3^ and MMA^+5^) amounts and CPM in the 13 donors analyzed. Values are log transformed. R = −0.46, p = 0.24.

The observation that extremely low and likely environmentally relevant concentrations of As^+3^ suppress T cell proliferation and activation of Th1 cells in susceptible individuals suggested that it might be important to measure arsenic exposures in our normal donors. Detectable levels of arsenic and its various inorganic (As^+3^ and As^+5^) and organic (MMA, DMA) forms were found in the urine of all donors collected as a first void on the day of the blood draw for HPBMC assessment (Table 2). In examining that mitogenesis data (Figure 1), high variability in the amount of T cell proliferation in control (no As^+3^ treatment) cultures among donors was noted. Urinary arsenic metabolites expressed on a µg/g creatinine basis. A correlation was performed for total arsenic (inorganic + organic), inorganic arsenic (As^+3^ and As^+5^), and organic arsenic (MMA^+5^, which also contains the MMA^+3^ fraction, and DMA^+5^, which also contains the DMA^+3^ fraction) in the urine for donors in Figure 1. We observed a weak inverse association between urinary MMA and the PHA-induced cell proliferation (r = –0.24, p = 0.2) in these donors (Figure 2).

**Table 2:** PHA-Induced^ 3^H-Thymidine Stimulation and Urinary Arsenic (Inorganic + Organic) in Normal Human Blood Donors.

		µg As/g Creatinine						^3^H-Thymidine	
Donor #	Creatinine mg/dL	AsB	As^+3^	DMA^+5^	MMA^+5^	As^+5^	Total As	CPMs	SD
BD205	166.00	2.15	0.23	6.07	0.59	0.18	11.14	35112	7585
BD211	91.20	ND	ND	3.91	0.31	0.00	4.69	10189	1962
BD203	56.30	ND	ND	5.59	ND	ND	5.40	31074	6357
BD223	57.30	2.03	ND	6.19	0.35	0.05	8.37	4280	389
BD224	95.90	ND	ND	5.45	0.64	0.48	6.31	15249	2409
BD225	219.00	0.02	0.72	4.82	0.91	0.50	7.30	3384	798
BD226	171.00	0.55	0.20	2.53	0.38	ND	3.87	13943	7026
BD227	274.00	7.91	0.41	5.30	0.40	0.12	15.88	6764	3915
BD228	45.10	0.16	ND	9.89	0.00	1.07	10.63	4539	3127
BD216	126.00	9.54	0.30	8.68	0.47	0.14	19.45	17338	5897
BD229	199.00	0.87	ND	3.01	0.09	0.01	3.78	15217	4242
BD230	274.00	1.00	0.53	2.61	0.60	0.29	4.91	20481	2832
BD231	83.80	ND	0.57	7.17	0.01	0.32	8.10	10657	2237
BD232	187.00	ND	0.21	3.71	0.48	0.19	4.64	4161	2424

Note: AsB = arseno betaine, MMA^+5^ and DMA^+5^ also include MMA^+3^ and DMA^+3^.

**Figure 3:**
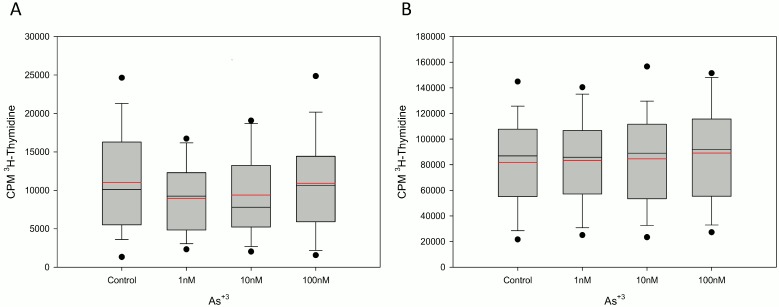
Proliferation response of PHA and anti-CD3/anti-CD28 stimulated PBMC treated with As^+3^. Comparison of PHA stimulated (A) and anti-CD3/anti-CD28 stimulated (B) T cell proliferation (^3^H-thymidine) for potential As^+3^ inhibition in 17 donors. Data shown are the Mean (red line) and Median (black line) ± SD with *p<.05.

Our original assessment of T cell proliferation induced by PHA was chosen because there are several epidemiologic studies published in the literature that indicate that PHA induced T cell proliferation is suppressed in individuals ingesting arsenic in drinking water [4], [5]. The stimulation of T cells via PHA is known to require monocytes [13], which have also been shown to be affected by As^+3^ at high concentrations [8], [14]. Therefore, it was of interest to compare the results for PHA stimulation with anti-CD3/anti-CD28 activation, which is monocyte-independent signal for T cells [13], [15]. The effects of As^+3^ on PHA and anti-CD3/anti-CD28 activation in 17 different individuals were compared (Figure 3). Although the magnitude of T cell proliferation (as evidenced by higher CPM ^3^H-thymidine) was increased in the anti-CD3/anti-CD28 stimulated cultures, there was no inhibition of As^+3^ at environmental levels (1–100 nM) in either the PHA or the anti-CD3/anti-CD28 treatment groups. Thus, As^+3^ at these low exposure levels is not suppressive to T cell proliferation in most individuals.

**Figure 4:**
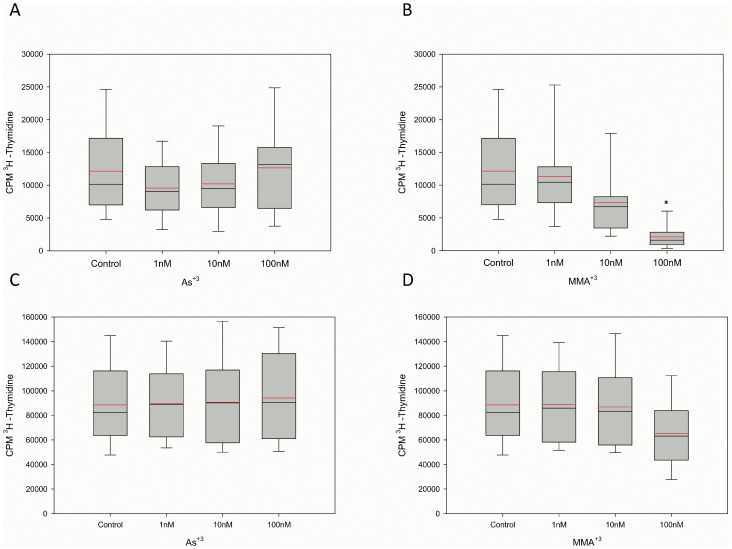
As^+3^ treated compared to MMA^+3^ treated PBMC stimulated with either PHA or anti-CD3/anti-CD28. Comparison of As^+3^ inhibition for PHA (A) and anti-CD3/anti-CD8 (C) and MMA^+3^ inhibition of T cell proliferation induced by PHA (B) and anti-CD3/anti-CD28 (D) in 8 donors. Data shown are the Mean (red line) and Median (black line) ± SD with *p<.05.

We have shown previously that MMA^+3^ is much more toxic than As^+3^ to murine bone marrow cells following *in vitro* exposures [3]. MMA^+3^ is formed, most notably in the liver and kidney, following *in vivo* ingestion of arsenic in drinking water and food. While HPBMC have extremely limited, if any, capacity to metabolize As^+3^ to organic forms *in vitro*, due to their poor expression of arsenite-3-methyl-transferase (AS3MT). A weak relationship between MMA and PHA-induced proliferation was observed (Figure 2). Therefore, the direct effects of MMA^+3^ on HPBMC T cell proliferation induced by PHA and anti-CD3/anti-CD28 were examined. MMA^+3^ and As^+3^ were compared at environmentally relevant exposure levels in 8 individuals. In agreement with earlier results, it was found that while As^+3^ at 1–100 nM concentrations did not suppress either PHA (Figure 4A) or anti-CD3/anti-CD28 (Figure 4C) induced T cell proliferation. However, MMA^+3^ caused a statistically significant suppression of T cell proliferation in the PHA-stimulated cultures at a concentration of 100 nM (Figure 4B).

## Discussion

Previous studies have shown that exposure of mice to 72 ppb arsenite for 30 days in drinking water suppresses a T-dependent antibody response in murine spleen cells [3]. This level of exposure was also found to be associated with an increased mortality in mice exposed to influenza virus [16]. Thus, the immune system appears to be a very sensitive target for arsenic exposures that may be associated with decreased host resistance to infectious agents. Epidemiologic studies have shown that exposure of humans to arsenic (As^+3^ and As^+5^) in drinking water may lead to suppression of T cell proliferation [4], [5]. However, there have been few studies that have examined the mechanisms responsible for immune suppression at environmentally relevant levels of exposures in human T cells. Numerous *in vitro* studies have been conducted on HPBMC, but most of these studies have examined extremely high concentrations of As^+3^ that may not be environmentally relevant. As^+3^ is known to produce oxidative stress at high concentrations (µM range) these concentrations of As^+3^ may actually stimulate proliferation and activation of many cells [17], [18], including T cells [19]. We focused on lower and more environmentally relevant concentrations of As^+3^ that would be expected from ingestion of contaminated foods and drinking water.

Because the immune system is mostly exposed to arsenic via the blood, when performing *in vitro* studies it is important to consider the actual blood levels of exposure to inorganic and organic forms of arsenic following ingestion of contaminated drinking water and food. Hall et al. [20] found that chronic ingestion of <50 ppb total arsenic in drinking water resulted in blood concentrations of 5–10 ppb arsenic (all forms). In this group, ∼50–200 µg total urinary arsenic/g creatinine was reported. A 10 ppb blood level of As^+3^ would equate to an *in vitro* concentration of 133 nM. The levels of exposure to arsenic reported in healthy donors in the present studies (Table 2) ranged from ∼4–20 µg total urinary arsenic/g creatinine, suggesting that ambient exposures in our studies would be estimated to be approximately 10% of that reported by Hall [20]. Therefore, the 10–100 nM exposure range used in the present studies for As^+3^ and other arsenic forms is considered to be environmentally relevant.

We found that the level of PHA-induced T cell proliferation in normal healthy donors was not associated with the total arsenic levels found in the urine obtained at the time of blood donation. The HPBMC from the majority of blood donors were not responsive to exposure to 1–100 nM As^+3^ *in vitro*. We did find that a small percentage (estimated at 5%) of people who were very sensitive to T cell inhibition by low As^+3^ (0.1–1 nM) exposures, an observation that was repeated on multiple occasions with blood draws over a period of more than a year. Thus, this appears to be a stable phenotype in these donors. While there were inadequate numbers of these susceptible donors, to fully investigate the genetic or other factors that determine their phenotype, it was possible to further define the sensitivity as it pertained to the activation of naïve CD4 T cells. It was determined that Th1 cells were very susceptible to low dose As^+3^ exposure, and perhaps the mixed lineage of Th17 and Treg cell precursors was also susceptible.

Most lymphoid cells lack the capacity to methylate arsenic due to the low expression of the arsenite-3-methyl-transferase (AS3MT) [10], [21], [22]. Therefore, it is important to understand the effects of organic (methylated) metabolites of arsenic formed in the liver and kidney because they contribute to blood levels found in humans. This was the rationale for examining the effects of MMA^+3^ on HPBMC in the present studies. We have shown that MMA^+3^ is more potent than As^+3^ in inhibiting mouse bone marrow and spleen cell function [3]. Therefore, we examined the effects of MMA^+3^ on T cell proliferation induced by PHA and anti-CD3/anti-CD28. Results showed that 100 nM MMA^+3^ inhibited T cell proliferation and that the PHA response may be more sensitive than the anti-CD3/anti-CD28 response. This is an intriguing finding as the PHA response requires the presence of macrophages [13], [15] which may also be sensitive to arsenic.

Thus, in summary, we found that that an understanding of arsenic metabolism and the formation of MMA^+3^ *in vivo* may be important for examining mechanisms of arsenic immunotoxicity. We also propose that the AS3MT status of individuals may play an important role in determining immunosuppression produced by environmental exposures to arsenic in human populations.
